# Extruded upper first molar intrusion: Comparison between unilateral and bilateral miniscrew anchorage

**DOI:** 10.1590/2177-6709.23.1.063-070.oar

**Published:** 2018

**Authors:** Mari Miura Sugii, Bruno de Castro Ferreira Barreto, Waldemir Francisco, Katia Regina Izola Simone, Ataís Bacchi, Ricardo Armini Caldas

**Affiliations:** 1 Universidade de Campinas, Faculdade de Odontologia de Piracicaba, Departamento de Dentística Restauradora (Piracicaba/SP, Brazil).; 2 Katholieke Universiteit Leuven, Mechanical Engineer School, Biomechanical Department (Leuven, Belgium).; 3 Faculdade de Medicina e Odontologia São Leopoldo Mandic, Centro de Pesquisas Odontológicas São Leopoldo Mandic, Departamento de Ortodontia (Campinas/SP, Brazil).; 4 IMED, Faculdade de Odontologia, Departamento de Prótese Dentária (Passo Fundo/RS, Brazil).; 5 Universidade de Campinas, Faculdade de Odontologia de Piracicaba, Departamento de Prótese e Periodontia (Piracicaba/SP, Brazil).

**Keywords:** Finite element analysis, Anchorage technique, Orthodontic

## Abstract

**Objective:**

The aim of his study was to evaluate the stress on tooth and alveolar bone caused by orthodontic intrusion forces in a supraerupted upper molar, by using a three-dimensional Finite Element Method (FEM).

**Methods:**

A superior maxillary segment was modeled in the software SolidWorks 2010 (SolidWorks Corporation, Waltham, MA, USA) containing: cortical and cancellous bone, supraerupted first molar, periodontal tissue and orthodontic components. A finite element model has simulated intrusion forces of 4N onto a tooth, directed to different mini-screw locations. Three different intrusion mechanics vectors were simulated: anchoring on a buccal mini-implant; anchoring on a palatal mini-implant and the association of both anchorage systems. All analyses were performed considering the minimum principal stress and total deformation. Qualitative analyses exhibited stress distribution by color maps. Quantitative analysis was performed with a specific software for reading and solving numerical equations (ANSYS Workbench 14, Ansys, Canonsburg, Pennsylvania, USA).

**Results:**

Intrusion forces applied from both sides (buccal and palatal) resulted in a more homogeneous stress distribution; no high peak of stress was detected and it has allowed a vertical resultant movement. Buccal or palatal single-sided forces resulted in concentrated stress zones with higher values and tooth tipping to respective force side.

**Conclusion:**

Unilateral forces promoted higher stress in root apex and higher dental tipping. The bilateral forces promoted better distribution without evidence of dental tipping. Bilateral intrusion technique suggested lower probability of root apex resorption.

## RESUMO

**Objetivos:**

o presente estudo teve como objetivo avaliar, por meio da Análise de Elementos Finitos, as tensões geradas por forças ortodônticas intrusivas em um molar superior e no osso alveolar circundante.

**Métodos:**

um segmento maxilar foi modelado no software SolidWorks 2010 (SolidWorks Corporation, Waltham, MA, EUA), contendo: osso cortical e medular, primeiro molar extruído, tecido periodontal e acessórios ortodônticos. Um modelo de elementos finitos simulou forças intrusivas de 4 N no elemento dentário, aplicadas a partir de mini-implantes em localizações distintas. Três diferentes vetores para mecânica de intrusão foram simulados: ancoragem em mini-implante vestibular, ancoragem em mini-implante palatino ou a associação de ambas. Todas as análises foram realizadas em termos de tensão mínima principal e deformação total. A análise qualitativa foi feita por meio do mapeamento da distribuição das tensões em gradiente de cores. A análise quantitativa foi feita em *software* específico para leitura e resolução de equações numéricas (ANSYS Workbench 14, Ansys, Canonsburg, Pennsylvania, EUA).

**Resultados:**

as forças intrusivas aplicadas simultaneamente nos dois lados (vestibular e palatino) resultaram em uma distribuição mais homogênea das tensões geradas, sem zonas de acúmulo de tensão e com uma resultante vertical para a movimentação intrusiva. As forças aplicadas somente em um lado, vestibular ou palatino, resultaram em zonas de concentração de tensão, com maiores valores, e na inclinação do elemento dentário para o lado em que a força foi aplicada.

**Conclusão:**

as forças unilaterais promoveram maior tensão no ápice radicular e maiores inclinações do elemento dentário. Já as forças bilaterais promoveram melhor distribuição das tensões e não resultaram em inclinação do elemento dentário. Assim, as forças intrusivas ancoradas bilateralmente apresentam menor probabilidade de reabsorção do ápice radicular.

## INTRODUCTION

Oral rehabilitation is a complex topic that frequently involves various specialists. This process may be hindered by the presence of malocclusions. Among malocclusions, dental supraeruption is one of the most complex conditions to be reversed.^1^

Supraeruption is derived from the lacking of antagonistic teeth and the absence of occlusal contact.^1^ Some different techniques have been described in the literature such as loops,^2^ transpalatal arch, extraoral devices^3^ and lately, the use of mini-screws.^1^ Another possibility to remove the occlusal interference is total crown preparation; however, when there is a substantial amount of tooth structure to be worn, previous endodontic treatment is necessary.^1^ ^,^ ^4^

The less invasive approach to restore occlusal plane is the orthodontic intrusion. In order to achieve better results with the intrusive forces, with low-risk of root resorption, steady anchorage associated with intermittent and light forces are required.^5^ ^-^ ^8^ Yet, it is important to consider some aspects such as: amount of alveolar bone surrounding the supraerupted tooth; conditions of adjacent teeth; periodontal health; and the required amount of intrusion, reported as determinant for choosing the intrusion method.^4^

Root resorption is an irreversible side effect of orthodontic treatment.^9^ Among the orthodontic movements tipping, torque, bodily movement into the lingual cortical plate of the maxilla,^10^ palatal expansion and specially intrusion, display greater risk of root resorption.^10^ ^-^ ^12^ Maxillary teeth are the most affected by external root resorption^5^ ^,^ ^13^ ^,^ ^14^ and molars have the second highest incidence, after incisors.^15^ So, the intrusion of the posterior segment must be planned and performed with prudence.

Conventional intrusion techniques for posterior teeth usually rely on multiple tooth anchorage. Based on reciprocal force mechanics, dental anchorage may result in undesirable movements and extrusion of other teeth rather than the aspired intrusion.^1^ Another approach for achieving intrusion is hight-pull headgear,^16^ which effectiveness is highly dependent on patient compliance.

Skeletal anchorage with mini-screws does not depend on multiple teeth involvement and have proven to be a minimally invasive and money-saving alternative.^17^ Moreover, this type of anchorage avoid undesirable tooth movements and does not dependent on patient cooperation, allowing better control of the applied force.^11^ Mini-screws are inserted in bone structure, and can resist to orthodontic displacement forces, preserving a static anchorage with no signs of marginal bone loss.^18^

*In-vivo* studies are not able to show the biomechanics inside the bone tissue, thus finite element analysis (FEA) has become a suitable method to investigate stresses under orthodontic forces.^19^

In orthodontics field, FEA has been successfully applied to assess stress distribution upon different amounts and directions of force.^9^ Results from FEA have shown great agreement with *in-vivo* results, therefore it may be considered a relevant and noninvasive method to virtually investigate stresses distribution during orthodontic force application.^20^

Thus, the purpose of this study was to determine, by using finite element analysis, the stress distribution and displacement effects of three types of mini-screw anchorage for a first upper molar intrusion: buccal, palatal or buccal-palatal. 

## MATERIAL AND METHODS

### Model construction

The model was designed based on a clinical situation of supraerupted maxillary first molar needing for orthodontic intrusion, with the help of design software SolidWorks 2010 (SolidWorks Corporation, Waltham, MA, USA). 

Maxillary models were constructed on the basis of cone beam computer tomography cross-sectional images from a posterior edentulous human maxilla. The maxillary segment was built considering a difference between cortical and cancellous bone. 

The geometric model of the tooth was constructed based on computed micro-tomography from an online database (Department of Oral Anatomy, School of Dentistry of Aichi-Gakuin University Available from: http://www.agu.ac.jp/english/index.html). The tooth model was created with distinction between enamel and dentin. Periodontal ligament was modeled considering a 0.3-mm thickness.^21^ ^,^ ^22^

**Figure 1:**
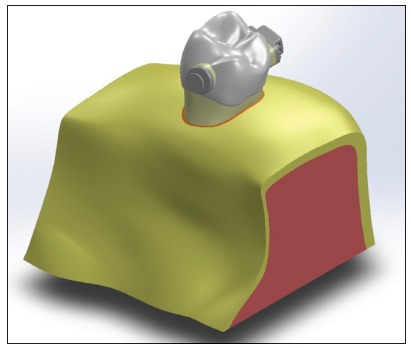
Complete template of the geometric model.

The Edgewise buccal tube and the orthodontic button (Morelli, Sorocaba, SP, Brazil) were modeled based on measurements with optical microscope at 40x magnification (VMM-100-BT; Walter UHL, Asslar, Germany) attached to a measurement device (QC 220-HH Quadra-Check 200; Metronics Inc., Bedford, USA). Full template is represented in Figure 1.

### Finite element analysis

After the modeling phase, the assembly was imported into the software ANSYS Workbench v. 14 (Ansys, Canonsburg, Pennsylvania, USA), for processing. Mechanical properties were used as described in Table 1. All materials were considered linearly elastic, homogeneous and isotropic. Mesh discretization was defined with tetrahedral 10-node elements. Model stability was performed using element quality analysis and the mesh was refined in regions of interest using specific tools from the software. All contacts were settled to Bonded. Thinned regions of the maxilla were considered as fixed constraints (zero degrees of freedom).

**Table 1:** Material properties.

Material	Young’s modulus (Gpa)	Poisson’s ratio
Cortical bone	13.7	0.30
Cancellous bone	1.37	0.26
Periodontal tissue	0.05	0.49
Enamel	80	0.25
Dentin	24.4	0.43
Orthodontic components (stainless steel)	193	0.30
Composite resin	22	0.27

**Table 2:** Highest compressive stress values (MPa).

	Force		
	Buccal and Palatal	Buccal	Palatal
Alveolar bone	0.105	0.374	0.24
Periodontal tissue	0.043	0.16	0.121
Dentin	0.401	0.657	1.07

**Figure 2:**
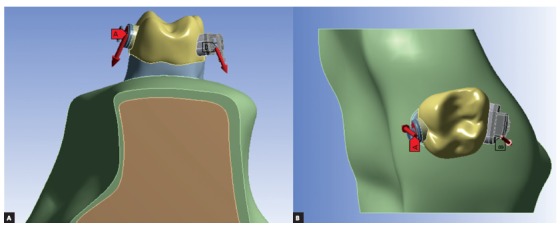
Orthodontic forces directed to mini-screw site: A) palatal force; B) buccal force.

Orthodontic intrusion forces were applied using three different techniques: 1) from the orthodontic double tube region to the buccal mini-implant; 2) from the palatal button to the palatal mini-implant and 3) combination of both forces simultaneously. A 4N force^1^ component (in total) was applied directed to the orthodontic mini screw site: unilateral directed to buccal mini-screw (4N) - located between first and second upper molar in buccal side; unilateral directed to palatal mini-screw (4N) - located between first and second pre molar in palatal side; and bilateral force, a combination of both directions (2N each) (Fig 2).

## RESULTS

Results were analyzed in minimum principal stress (dentin and bone) and total deformation (periodontal ligament). Minimum principal stress was used to obtain better compressive stress analysis (negative values), and total deformation was used to predict the resultant movement of the intrusive forces.

**Figure 3:**
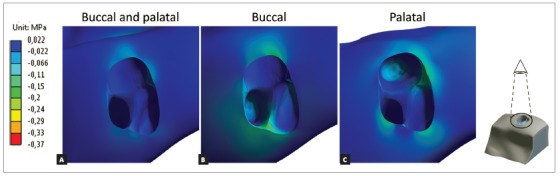
Minimum principal stress at alveolar bone. Negative values means compressive region. Occlusal view of dental alveolus, tooth was hidden for better visualization.

Figure 3 shows the minimum principal stress in alveolar bone. Bilateral forces presented good stress distribution between cortical and cancellous bone and lowest compressive values, equally distributed (similar values) for all three periapical regions. Buccal force generated a compressive region at buccal furcation and both buccal roots. Palatal force generated compressive region near mesial furcation and periapical region of palatal root.

**Figure 4:**
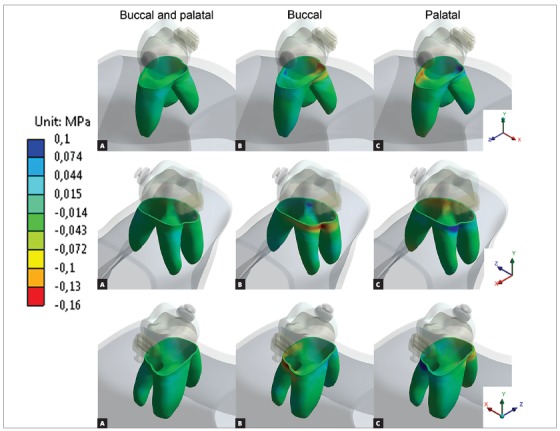
Minimum principal stress at periodontal ligament. Negative values represent compressive regions.

**Figure 5:**
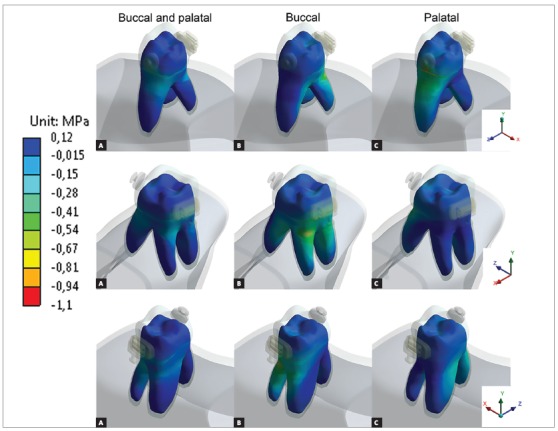
Minimum principal stress at dentin. Negative values represent compressive regions.

The periodontal ligament (Fig 4) presented similar behavior as previous results,^19^ ^,^ ^30^ with well distributed compressive stresses when bilateral forces were used. Single-sided forces presented highest compressive values, especially near cancellous bone and periapical region of the same side force was applied. Higher values were found in dentin (Fig 5) near cortical bone at the same side of force application, without high compressive values in periapical region.

**Figure 6:**
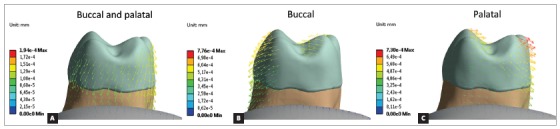
Tooth movement: arrows indicate movement direction and magnitude.

Figure 6 show that bilateral forces resulted in predominance of vertical vectors (intrusive resultants), single forces presented oblique vectors, with vertical resultants and lateral resultants. Also, higher values of deformation were found for unilateral forces, compared to bilateral force.

## DISCUSSION

Dental intrusion is considered one of the most challenging movements in orthodontics. Posterior teeth intrusion is even more complex due to its roots anatomy and lack of anchorage. In order to prevent undesirable movements and root resorption, the degree and direction of force must be controlled.^23^

The present study evaluated by finite element analysis the stress distribution in a maxillary first molar and alveolar bone after simulation of an intrusion force. Forces magnitudes were chosen accordingly to a systematic literature review about skeletal anchorage.^18^

Minimum principal stresses increased on the alveolar bone, periodontal ligament and dentin, near dentin-enamel junction (DEJ), on the side in which the intrusive force was applied (Figs 3, 4 and 5). This observation suggests a possible dental inclination directed to the side to which the force was applied, as presented in dislodgement analysis (Fig 6). Furthermore, it can be inferred that intruding molars can enhance risk of gingival retraction near DEJ, since bone is prone to reabsorb during constant compressive forces at adjacent periodontal tissue. Çifter and Saraç^19^ found buccal tipping in molars when an intrusive force was applied; also, an uniform stress distribution when forces were applied from buccal and palatal sites concomitantly, these results are in agreement with the present findings. 

Palatal intrusive force caused higher compressive stresses near mesial furcation of palatal root and around root apex on alveolar bone, near DEJ of periodontal ligament and on cervical dentin. Compressive stresses originated from tipping movement, as in unilateral palatal force, are known to cause more severe root resorption than bodily movements.^24^ This is due to the distribution of stresses per area unit, which is more concentrated in smaller apex area than in cervical region. It is also important to emphasize that the more tipping vectors are present on intrusion mechanics, the less periodontal ligament can soften stresses.^25^

Single buccal force has caused higher compressive stresses on vestibular furcation and on alveolar bone around vestibular roots apex, on cervical region of periodontal ligament and on the cervical third of the roots dentin. When considering forces applied to buccal side, their resulting stresses are distributed in two roots instead of only one, as in the case of the palatal root. Also, is already known that unilateral forces promote tipping movement and stress concentration around buccal cervical regions.^26^ However, this is not a major concern considering the larger area and the slight deflection of the bone crest.^25^

In addition, bilateral forces showed better stress distribution in alveolar bone and the roots. This type of movement makes the force perpendicular to dental alveolus, resulting in stresses efficiently softened and distributed along periodontal ligament. Also promoted a more efficient intrusion movement, with higher vertical resultant vector than the other techniques.

Resorption as a side effect of orthodontic intrusion has been reported for years. Some studies showed that intruding incisors lead to stress accumulation at root apex.^27^ ^,^ ^28^ There are different degrees of severity: 1) Resorption of outer cemental layers, which can be fully regenerated; 2) Besides the outer cemental layers there is dentin resorption, usually repaired with cementum material; and 3) circunferencial apical root resorption, on this stage root shortening is evident.^12^

When intrusion forces are applied, the periodontal ligament is damaged, creating a sterile necrotic tissue (hyalinization tissue) and consequently causing macrophage-like cell activation and also differentiation of osteoclasts not fully expressed, into fully developed ones. The sterile necrotic tissue is then slowly removed by these cells, starting at the region of blood supply to the periodontal ligament. As the root surface is very close to this area, the outer layers of cementoblasts and cementoids can be damaged too. Resorption process goes until the necrotic tissue is no longer present, creating bone lacunae and decreasing the pressure exerted through force application.^12^ ^,^ ^25^ ^,^ ^29^ Therefore, the heavier applied force, the longer the resorption process takes place, enabling more root damage. Techniques which lead to a lower stress induction in periodontal ligament associated to tooth correct movement is desirable, possibly promoting a lower dentin resorption.

Despite this fact, this FEA study revealed stress accumulation in tooth furcation rather than in root apex, suggesting that molars are less likely to apical root resorption when compared to single-rooted teeth during intrusion forces. Jeon et al,^30^ also using FEA have found that molar intrusion displayed stress zone at furcation and along root length. According to these authors, the rationale consists on the different number of roots and consequently on the difference in total root area between single and multi-radicular teeth. Stresses are better spread along three roots rather than in only one.

As in any other hypothetic simulation, there are some limitations inherent to Finite Element Analysis. There are individual variations, especially because of differences in teeth anatomy and dimensions, between patients. Also, stress distribution and interaction between hard and soft tissue are complex.^9^ Therefore, creation of an exact mathematic model for each individual case is unlikely.^19^ Still, even with these disadvantages, this study showed great convergence with other FEA investigations, *in vitro* research and clinical findings cited on the literature.

## CONCLUSION

This FEA simulation could provide the following conclusions: Unilateral force unleashed higher stress in root apex and higher evidence for dental tipping directed to mini-implant site; the bilateral force promoted a more homogeneous stress distribution without evidence of dental tipping. Bilateral intrusion technique suggested a vertical movement of intrusion and lower probability of root apex resorption.
